# Femoral Nerve Palsy Following Direct Anterior Total Hip Arthroplasty in a Patient With Iliac Venous Stents: A Case Report

**DOI:** 10.7759/cureus.86799

**Published:** 2025-06-26

**Authors:** Thomas S Peacock, Beruk Sherif, Ji Won Lee, Gregorio Baek, Henry Boucher

**Affiliations:** 1 Orthopaedics, Georgetown University School of Medicine, Washington, D.C., USA; 2 Orthopaedics, Union Memorial Hospital, Baltimore, USA

**Keywords:** adverse event, anterior approach, femoral nerve palsy, femoral triangle, hip and knee replacement, iatrogenic complication, iliac vein stent, primary hip replacement

## Abstract

A male in his 50s with chronic venous insufficiency and bilateral iliac venous stents who had undergone right direct anterior approach total hip arthroplasty presented on postoperative day one with sensory deficiencies and motor weakness in the distribution of the right femoral nerve. The patient was diagnosed with severe femoral nerve palsy, based on absent quadriceps activation and electromyography-confirmed denervation. Though a known complication of this approach, severe femoral nerve palsy is a rare occurrence. The authors hypothesize that venous stents may have contributed to the increased vulnerability of the femoral nerve during retraction. Great care should be taken in patients with prior stenting to avoid further compression perioperatively and subsequent development of femoral nerve palsy.

## Introduction

With over 450,000 cases performed annually and up to 93% of patients reporting improvement in mobility and pain, total hip arthroplasty (THA) is a common and successful treatment for end-stage osteoarthritis [1]. Among different surgical approaches, the direct anterior (DA) approach has become a dominant technique for THA in North America in recent years. Currently, over 50% of surgeons performing THAs complete more than 100 DA approach THAs per year [2]. Compared to the posterior and lateral approaches, the DA approach does not release any musculature [3,4] and is associated with a quicker recovery, shorter hospital stay, and decreased risk of dislocations [3]. Limitations of the DA approach include a significant learning curve and a surgical technique that predisposes the lateral femoral cutaneous nerve to injury. The learning curve for a DA THA is particularly pertinent, as complication rates are twice as high in the beginning stages of the surgeon’s career compared to when one reaches proficiency, with shorter operative times at 100 cases [5]. In addition, prolonged retraction of the anterior proximal thigh and pelvic structures places the femoral neurovascular structures at risk. These factors potentially lead to complications such as femoral nerve palsy (FNP) [3,4].

Though rare, the prevalence of FNP varies by approach and is a cause of great morbidity. FNP is most prevalent in the DA approach with reported rates of 0.4% to 1.1% [6,7]. Furthermore, FNP leads to functional limitations that may affect short- and long-term outcomes. With FNP, patients are limited in using their affected leg, which subsequently affects activities of daily living such as getting dressed, bathing, and doing chores. Furthermore, FNP leads to decreased knee range of motion and stability, which may contribute to falls in the postoperative period [8]. Falls are a serious cause of morbidity for patients in this vulnerable period, leading to serious complications requiring revisions due to dislocation and periprosthetic fracture [9]. These issues all potentially contribute to poor short and long-term outcomes and related dissatisfaction after THA.

The risk factors for FNP include female sex, short stature, and poor placement of the anterior acetabular retractor [7,10]. Although not investigated by previous studies, iliofemoral stents may be another contributing risk factor for FNP, which are used frequently to treat May-Thurner syndrome, chronic deep venous thrombosis (DVT), or other vascular pathologies [11,12]. As the usage of iliofemoral stents in the management of chronic DVT increases, the incidence of venous stents in the general populace may also increase [12,13]. Iliofemoral stents, by nature of their rigid design, may reduce the natural compressibility of vascular structures within the femoral triangle. The proximity of the femoral vein is rarely an issue during DA approach THA because of the compressibility of the vein during retraction. However, the presence of an iliofemoral stent may be problematic because it limits vein compressibility during retraction, allowing for pressure on the femoral nerve as it is compressed between the retractor and the solid stent in the femoral vein. Currently, there is a lack of literature that investigates the presence of an iliofemoral stent as a risk factor for the postoperative development of FNP in patients undergoing the DA approach THA.

In our case study, we aimed to fill this knowledge gap. We present the case of a patient with a history of iliofemoral stents who developed FNP after the DA approach THA. Consent was obtained from the patient to present his case in this manner. We propose that the presence of an iliofemoral stent may have created anatomical conditions that increased the risk of femoral nerve compression during retraction. This has implications for clinical practice because as the aging population with multimorbidity grows [14], the number of patients with iliofemoral stenting will increase, putting these populations at risk for complications such as FNP. Surgeons should be cognizant of this complication, which may lead to significant morbidity and poor postoperative outcomes after the DA approach THA.

## Case presentation

**Figure 1:**
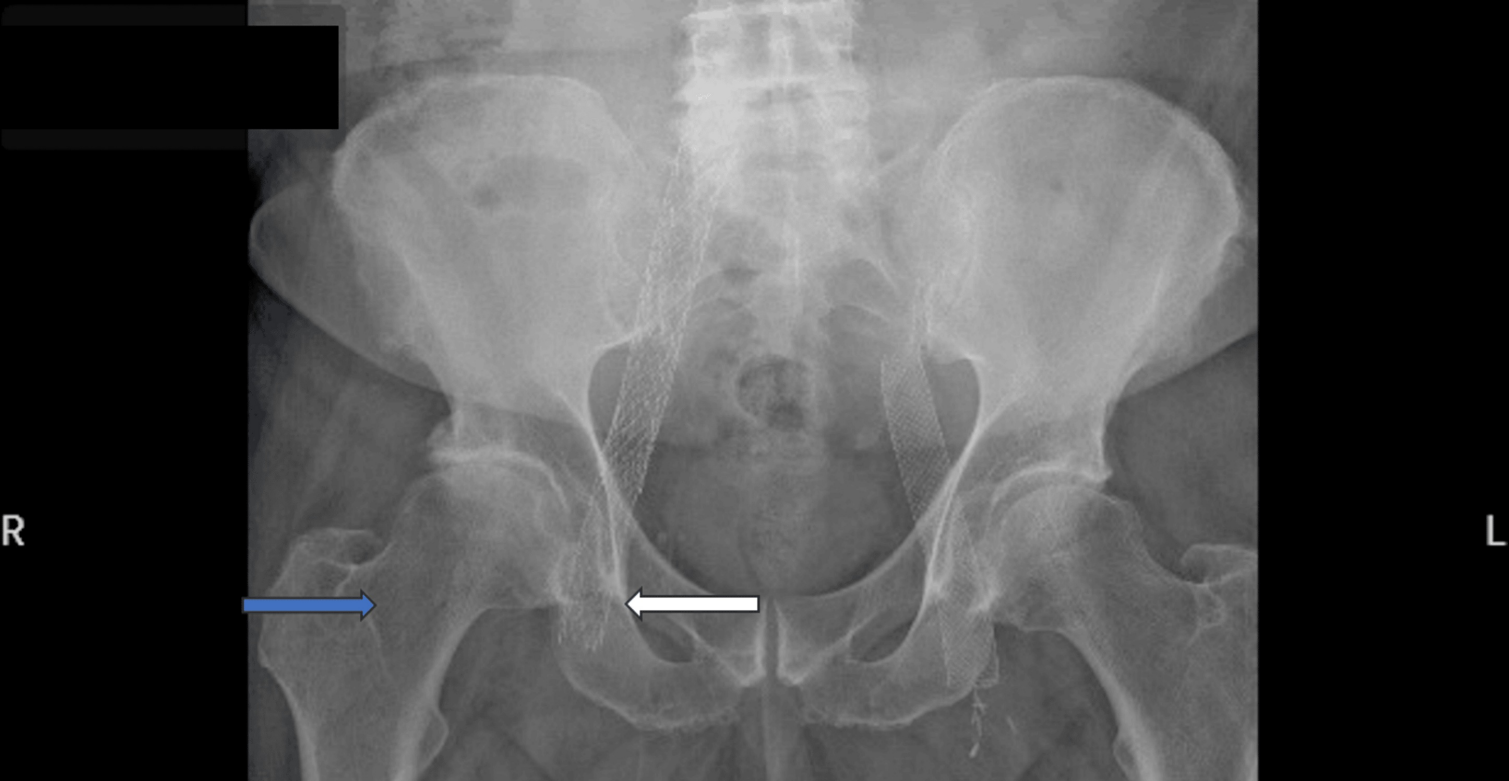
X-ray showing the proximity of the iliac venous stent (white arrow) to the surgical site (blue arrow).

A male in his 50s with severe hip osteoarthritis and intractable pain despite conservative treatment presented to the clinic for surgical treatment. At baseline, the patient was ambulatory without assistive devices and had no focal neurologic deficits. Relevant past medical history included hypertension, obesity, and chronic DVTs. The patient was treated for bilateral DVTs and pulmonary embolism two years before his THA and had a bilateral thrombectomy and placement of a bilateral venous stent one year before the surgery. He was placed on Xarelto indefinitely. He was cleared by vascular surgery for a right THA. X-ray showed bilateral osteoarthritis with the presence of bilateral venous stents in the femoral veins (Figure 1).

The patient underwent a right DA approach THA. There were no intraoperative complications. Surgical exposure was adequate and achieved without technical difficulty. The total operative time was 58 minutes, which falls within the surgeon’s average range [5]. Immediately postoperatively, the patient complained of numbness extending from his distal thigh to the anteromedial leg and the inability to lift the lower extremity. The next day, he reported poor control of the right quadriceps and increasing numbness. He was discharged with a hinged knee brace locked in extension to stabilize his gait. Despite this intervention, the patient sustained a fall on postoperative day one due to quadriceps weakness while changing clothes without the knee brace; no additional injuries were sustained.

At his first follow-up office visit three weeks postoperatively, he had 0 out of 5 strength with knee extension, but he was able to actively plantar flex and dorsiflex his ankle. He was also able to actively flex his hip with a flexed knee. With the clinical presentation consistent with FNP, the surgeon ordered an electromyogram (EMG) at the three-week postoperative follow-up, which confirmed a right femoral and saphenous nerve conduction failure and no voluntary activity of his quadriceps muscles, consistent with a severe right FNP (Table 1). Postoperative MRI revealed mild nerve edema without evidence of mass effect or hematoma, supporting a compressive neuropathy etiology rather than structural impingement.

**Table 1:** Electromyogram results indicating severe acute right femoral neuropathy consistent with femoral nerve palsy. Increased spontaneous activity and absence of voluntary motor unit recruitment in the quadriceps muscles are noted.

Electromyogram summary table									
	Spontaneous					Motor unit action potential			Recruitment pattern
	Insertion activity	Fibrillation	Positive sharp waves	Fasciculations	High frequency	Amplitude	Duration	Polyphasic potential	
Right vastus medialis	3+	3+	3+	None	None				No activity
Right vastus lateralis	3+	3+	3+	None	None				No activity
Right tibialis anterior	N	None	None	None	None	N	N	N	N
Right gastrocnemius (medial)	N	None	None	None	None	N	N	N	N

The patient continued with physical therapy with an emphasis on nerve reeducation, use of a hinged knee brace locked in extension, and an assistive device. At four months, he sustained two more falls as he independently tried to transition from the knee brace to only a cane, without additional injury. As a result, he continued with the brace after the second fall. At his six-month visit, he reported significant improvement in strength. He was able to go up and down stairs with the assistance of a railing and began to unlock his knee brace at the direction of his therapist. At his nine-month postoperative office visit, he continued to gain strength in his leg, with a strength of 4 out of 5 with active right knee flexion. At the one-year follow-up, the patient had full return of strength, no activity limitations, and no pain in the hip region, but mild, persistent subjective numbness in the medial knee and leg. He had overall improvement in his hip function compared to before the surgery, with a hip disability and osteoarthritis outcome score of 39.90 preoperatively and 76.78 at one year, which exceeded the minimal clinically important difference for this instrument (7.76), suggesting meaningful functional recovery [15]. He also had an improvement in overall mental and physical health with 12-Item Short Form Survey (SF-12) scores of 51.66 and 23.11 for mental and physical health, respectively, preoperatively and 57.26 and 37.22, at his one-year follow-up.

## Discussion

FNP following THA is a rare complication that affects sensory and/or motor function. It presents as symptoms of femoral neuropathy, such as numbness and tingling, and/or severe motor weakness and dysfunction [6]. These sensory and/or motor deficits may contribute to a fall, which is a major risk factor for dislocation and periprosthetic fracture [9]. Furthermore, these complications may lead to long-term functional issues and poor patient satisfaction after THA. Diagnosis of FNP is mostly clinical; however, a spinal MRI or EMG may be indicated to rule out a spinal cause or hematoma. In our case of a male in his 50s with a relevant history of chronic DVTs treated with iliofemoral stenting undergoing a DA approach THA, symptoms presented immediately after surgery, with a fall occurring directly after discharge on postoperative day one. FNP with quadriceps paralysis was confirmed by EMG at five weeks postoperatively, and the patient remained significantly functionally limited for six months postoperatively. He began regaining significant strength and sensation after six months and continued to improve. His last postoperative visit to date was at one year, and he showed resolution in strength and motor function deficits, despite some remaining lower extremity numbness.

In the DA approach THA, the causes of FNP can be attributed to several different factors, including the surgeon’s experience with DA approach THA, learning curve, poor placement of the anterior acetabular retractor, and the patient’s anatomy (i.e., short stature) [5,7]; these factors potentially increase the risk of femoral nerve entrapment. We present here a distinct but possible contribution to FNP that presented in this patient, which has not been previously described in the literature. The presence of a venous stent in the patient’s femoral vein provided an incompressible surface in the femoral triangle, which may have contributed to a crushing injury to the nerve during retraction. Though we cannot prove a causal link, the circumstances in this case raise the question of the iliofemoral stent as a risk factor for FNP.

**Figure 2:**
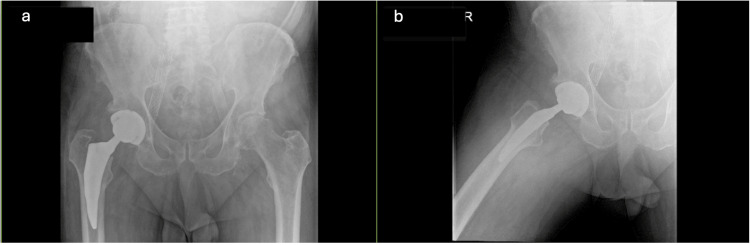
Postoperative anteroposterior (a) and lateral (b) view X-rays of the pelvis showing good prosthetic placement without evidence of malposition or loosening.

The surgery was performed by a high-volume arthroplasty surgeon with more than 10 years of experience using the DA approach, reducing but not eliminating the potential for technical complications. In addition, there was no evidence of prosthetic malposition or loosening postoperatively (Figure 2). Given the lack of prior FNP complications with this surgeon and shared decision-making with the patient, the surgeon proceeded with the DA approach. We are still unable to prove that errant retractor placement was not a factor, which is a limitation of our report.

In patients who experience FNP after THA, there is expected recovery of motor function within two years, although sensory dysfunction may continue after that timeline [7]. As indicated in our report, despite early diagnosis and treatment of FNP in our patient, there were several postoperative falls, which stresses the importance of patient education to ensure that the patient adheres to the postoperative protocol to mitigate fall risk. As expected, the patient had excellent pain relief from the hip arthroplasty, which may have led to a lack of compliance with brace use, which led to multiple falls. Fortunately, none of the falls led to further complications in this case, as his patient-reported outcome (PRO) surveys showed an improvement in both his hip pain and overall mental and physical health at one-year postoperatively compared to preoperative PROs.

## Conclusions

While it may not be entirely possible to eliminate the risk of FNP in patients undergoing THA with a prior ipsilateral iliofemoral stent, surgeons should remain vigilant about this potential complication. Early identification, thorough evaluation, and timely management of FNP are essential to minimizing adverse outcomes such as postoperative falls, which can significantly hinder recovery. Preoperative counseling should include a discussion of the potential increased risk of FNP in patients with iliofemoral stents, particularly when considering the DA approach. Alternative surgical approaches, such as the posterior approach, may be considered in select cases, as they may pose a lower risk of femoral nerve traction. If the DA approach is pursued after shared decision-making, the surgical team should be prepared to monitor for signs of neuropathy, implement fall-prevention strategies, and initiate appropriate rehabilitation to support functional recovery and optimize postoperative outcomes.
